# The Role of Short-Chain Fatty Acids in Mediating Very Low-Calorie Ketogenic Diet-Infant Gut Microbiota Relationships and Its Therapeutic Potential in Obesity

**DOI:** 10.3390/nu13113702

**Published:** 2021-10-21

**Authors:** Naser A. Alsharairi

**Affiliations:** Heart, Mind & Body Research Group, Griffith University, Gold Coast, QLD 4222, Australia; naser.alsharairi@gmail.com

**Keywords:** VLCKD, infant gut microbiota, obesity, adipose tissue, SCFAs, pregnancy, lactation

## Abstract

As the very low-calorie ketogenic diet (VLCKD) gains increased interest as a therapeutic approach for many diseases, little is known about its therapeutic use in childhood obesity. Indeed, the role of VLCKD during pregnancy and lactation in influencing short chain fatty acid (SCFA)-producing bacteria and the potential mechanisms involved in the protective effects on obesity are still unclear. Infants are characterized by a diverse gut microbiota composition with higher abundance of SCFA-producing bacteria. Maternal VLCKD during pregnancy and lactation stimulates the growth of diverse species of SCFA-producing bacteria, which may induce epigenetic changes in infant obese gene expression and modulate adipose tissue inflammation in obesity. Therefore, this review aims to determine the mechanistic role of SCFAs in mediating VLCKD-infant gut microbiota relationships and its protective effects on obesity.

## 1. Introduction

The VLCKD is distinguishable from other diets because of its very low carbohydrates (CHO), high fat and moderate protein intake [1]. It typically comprises of 70% fat, 20% protein and 10% CHO (<50 g per day) [1]. The VLCKD induces ketosis [1,2,3,4], a metabolic state which increases the production of ketone bodies (KBs) in the liver [5]. The VLCKD favours animal/plant-derived fat and protein intake, from sources such as nuts and seeds, butter, cheese, cream, beef, lamb, chicken, olive and fish oil [6]. Such a diet is superior to low-CHO diets (LCDs) and low-fat diets (LFDs) in producing sustained ketosis and improving metabolic markers [7,8,9]. The VLCKD can be considered an effective dietary approach for weight loss [10]. However, it is not totally safe and has sparked controversy for its long-term effects [11]. The VLCKDs proposed protocol for successful weight loss is divided into three phases. These include the active phase (VLCKD 600–800 kcal/day, 80% of target weight), the re-education phase (LCD 1200–1500 kcal/day, 20% of target weight) and the maintenance phase (balanced diet 1500–2200 kcal/day) [12]. The VLCKD can maintain the body in the state of ketosis by increasing levels of acetoacetate (ACA) and β-hydroxybutyrate (βOHB) in the blood, which are the two major KBs used as energy sources [13].

Microbiota-derived SCFAs, primarily acetate, propionate and butyrate, are metabolites produced by gut microbiota via dietary non-digestible CHO fermentation [14,15]. Butyryl-CoA: acetate CoA-transferase is the major pathway that exists in the formation of butyrate. Certain bacteria of the families *Ruminococcaceae* and *Lachnospiraceae* within the phylum *Firmicutes* utilize the pathway via butyryl-CoA dehydrogenase (BCD) to convert acetyl-CoA to butyryl-CoA and subsequently to butyrate [14,15,16,17,18]. SCFAs play a significant role in CHO and lipid metabolism. Butyrate and acetate are used as precursors for lipid synthesis (cholesterol, long-chain fatty acids), whereas propionate is used as precursor for hepatic gluconeogenesis [14,19]. Lactate is an organic acid produced by *Bifidobacterium* and lactic acid bacteria (LAB), which acts as an intermediate metabolite and a substrate for butyrate formation [19,20].

There is a potential synergistic effect of butyrate with VLCKD in inducing ketosis [21]. βOHB is produced in the liver from free fatty acids (FFAs) during fasting or starvation and serves as a transporter of fuel to peripheral tissues [22]. βOHB and butyrate share a degree of function and structure similarity. They have a wide range of cellular signalling roles, including regulate gene expression by epigenetic modifications, lipid metabolism and gut homeostasis [22,23,24], and their actions have therapeutic potential in many diseases such as obesity [25] and asthma [26].

The gut microbiota is a critical component during pregnancy and lactation where maternal diet may influence both the mother’s and the infant’s gut microbiota diversity and richness [27,28]. A well-planned diet including a variety of protein-rich plant foods, dietary fibre and omega-3 polyunsaturated fatty acid (PUFA) during pregnancy and lactation is recommended [29], which may tend to produce high amounts of fecal SCFAs by SCFA-producing bacteria [27,30]. Maternal intake of protein, high fat and omega-3 PUFA may influence the infant gut microbiota through the epigenetic mechanisms for histone acetylation and DNA methylation [31,32]. Maternal gut microbiota and its metabolites, in which SCFAs are the major products generated by the fermentation of microbiota-accessible carbohydrates (MACs), may exert regulatory effects on host energy metabolism [33,34] and the infant immune system [33,35,36]. Breast milk constitutes the main source of seeding microbes in the neonate gut [37]. It plays a key role for vertical transmission of microbes from mother to infant via the entero-mammary pathway. This route proposes that microbiota can be transferred from the maternal gut lumen by dendritic cells (DCs) to the mammary glands through the blood/lymphatic systems, and then move to the newborn and subsequently colonize the gut [38,39]. SCFA-producing gut bacteria have the ability to stimulate SCFA production in breast milk via the systemic circulation [40], which in turn enters the infant intestinal tract through the breast milk [26].

Childhood obesity has become one of the most significant global health challenges over the last decades [41]. Unfortunately, the prevention of obesity may need to be addressed at its origin, which is complex and multifaceted with no single factor domain as a determinant. The range of contributing factors comprise epigenetics, genetics, parental/infant body mass index (BMI), smoking during pregnancy, early antibiotic use, birth by caesarean section, an unhealthy diet, formula feeding and microbiota [42,43,44,45,46,47]. Among these, the infant gut microbiota has received great interest in the past few years. Infancy is a key period in the development of the gut microbiota, with the colonization rate of commensal species increasing after birth [18]. However, obesity influences gut microbial diversity and composition and may lead to dysbiosis, which dysregulates metabolic homeostasis [48]. Gut microbiota composition varies greatly between obese and non-obese infants. Epidemiologic evidence from infant studies has demonstrated lower proportions of SCFA-producing bacteria such as *Bifidobacterium* and *Bacteroides* spp. in the gut microbiota of obese children compared to lean counterparts [42]. Furthermore, infants of obese mothers have significant alterations in their gut microbiota composition, which may lead to a later-life obesity risk. The transmission of obesogenic microbes from mother to infant has the greatest potential for childhood obesity, with a higher abundance of fecal *Lachnospiraceae* (e.g., *Coprococcus*, *Ruminococcus*) in vaginally and emergency cesarean-delivered infants which mediated the association between maternal overweight/obese and childhood obesity at ages 1 and 3 years [49].

Adipose tissue (AT) is known to be the main contributor to immune dysregulation, metabolic diseases and low-grade chronic inflammation during obesity [50]. AT macrophages represent the major component of C-C motif chemokine ligand-2 (MCP-1) and tumor necrosis factor-alpha (TNF-α) in AT and upregulate of Interleukin (IL-6) expression, where they can induce inflammatory changes in adipocytes [51]. Pregnancy and early infancy are critical periods of increased oxidative stress (OS) and pro-inflammatory cytokines levels [52,53,54,55]. OS plays a significant role in the development of obesity and its related diseases, in which the role of dysfunctional AT is involved [56]. OS results from the shift in the balance between the reactive oxygen species (ROS)-generating systems (e.g., nitric oxide synthase) produced by mitochondria and the capability of the antioxidant system to detoxify them [57].

A high-fat maternal diet during pregnancy has been shown to cause dysbiotic gut microbiota in infants, which has been linked to obesity, leading to developmental programming that can contribute to obesity-associated chronic inflammatory diseases. Additionally, maternal high-fat diet-induced obesity during lactation may alter breast milk microbiota composition, which may in turn contribute to infant gut dysbiosis and increase obesity susceptibility later in life [27]. While an obesogenic diet or obesity during pregnancy and lactation have a significant influence on the infant gut microbiota changes, human studies linking these alterations with an increased risk of childhood obesity are controlled for potential maternal life factors such as mode of feeding, antibiotic use and mode of delivery [27]. Therefore, a better understanding of how gut dysbiosis might induce obesity in early life is needed. Indeed, the mechanisms by which VLCKD could modify obesity risk in early life remain to be understood. The maternal VLCKD composition during pregnancy and lactation may influence the infant gut SCFA-producing bacteria [26], which play key roles in regulating glucose homeostasis, appetite, inflammatory response and the immune system [58,59]. Nutritional ketosis induced by VLCKD has a suppression effect on hunger and appetite [60], in which appetite-regulating gut hormones promote weight loss, increase circulating FFAs, reduce food intake and regulate energy homeostasis [60,61], through an increase of hypothalamic malonyl-CoA cellular levels [62]. The VLCKD during pregnancy and lactation may include olive oil, coconut oil, butter, cream cheese, sour cream, eggs, fish, lamb, ham, beef, poultry, low-CHO nuts and non-starchy vegetables [63,64,65], which are potential sources of dietary fibre, protein, polyphenols, saturated fatty acid (SAT), monounsaturated fatty acid (MUFA) and PUFA. SCFAs are involved in the mechanism linking the VLCKD during pregnancy and lactation to the infant gut microbiota, which may modulate allergic asthma in infants [26]. Given the fact that SCFAs influence obesity-related asthma [66], it is perhaps the case that SCFAs from VLCKD-infant gut microbiota interactions may have potential therapeutic implications for reducing obesity.

## 2. Methods

This non-systematic review aims to explore the mechanisms by which microbiota-derived SCFAs mediate the VLCKD-infant gut microbiota relationship and its therapeutic efficacy in reducing obesity. To achieve this, a literature search was carried out using the PubMed/MEDLINE database to retrieve English language studies in humans in the past 20 years. The author has independently identified the most relevant studies including randomised controlled trials (RCTs), experimental and observational, and reviews/systematic reviews. The following keywords were used to search for articles: obesity, AT, infant gut microbiota, gut inflammation, epigenetic, SCFA, KD, KBs, pregnancy and lactation.

## 3. Metabolic Adaptations in Pregnancy and Lactation: Ketone Body Metabolism 

Metabolic adaptations in pregnancy and lactation are essential for fetus growth and development. Body fat appears to change significantly during pregnancy. Abdominal AT (AAT), including subcutaneous, visceral and total AAT, increases during the first half of pregnancy, which is associated with increased risk of gestational diabetes mellitus during the last trimester [67]. Placental growth hormones such as lactogen, progesterone and prolactin are implicated in increased appetite through activating leptinsecretion, β-cell expansion and insulin dependent lipogenesis, leading to enhance maternal/fetal fat deposition, fetal growth and glycogenesis [68,69]. Hyperphagia in mid and late-pregnancy may occur as a result of leptin resistance, which may contribute to increased body fat deposition in order to be used for metabolic demands in lactation [69,70]. Low leptin levels are indicative of leptin resistance in newborns, in which low cord blood leptin levels have been shown to be associated with increased BMI growth trajectories in infants of overweight/obese pregnant women [71].

Lactation is characterized by metabolic changes such as hyperphagia, accompanied by significant changes in hormones, which are controlled by the hypothalamus to meet the energy demands of the infant [72]. Prolactin, a key lactogenic hormone produced by pituitary lactotrophs, plays a significant role in inducing changes in milk composition and increasing milk production and oligosaccharide concentrations. However, prolactin has no significant changes in milk protein, fat or nutrient levels [73]. Appetite-regulating hormones/adipokines such as leptin and adiponectin, which are known to be in higher levels in breast milk, have been found to influence the infant serum of these hormones [74,75]. Leptin is proposed to have a lipophilic nature as it exists in whole milk [76], is implicated in the neonatal T-cell immune response [77], regulates glucose homeostasis, food intake programming in infants [78,79], and is correlated with maternal BMI [55,80]. Interestingly, high levels of leptin in exclusively breastfed infants born to obese mothers correlate with the metagenomics pathway of the infant gut microbiota that may reduce gut inflammation and enhance the gastrointestinal barrier [81]. Adiponectin synthesis by adipocytes has been found to regulate glucose/lipid metabolism and food intake in infants [77,78,79], and correlates with longer gastric emptying time in term breastfed infants [82]. Other adipokines such as resistin and ghrelin have been implicated in food intake regulation, energy balance and glucose homeostasis, suggesting a potential role of these hormones in enhancing infant growth and development [77,78,79]. Studies investigating the association between breast milk adipokines and weight gain in infants are conflicting. Leptin, adiponectin and ghrelin may increase/decrease weight gain or increase fat mass in infants. In contrast, no association was noted between resistin and weight gain. Breast milk adipokines dysfunction/dysregulation may contribute to obesity later in life [80]. Overall, adipokine action in the hypothalamus regulates hunger/satiety and glucose homeostasis, thus potentially improving weight reduction in infants.

Being in a state of nutritional ketosis (blood βOHB level of 0.5–3.0 mmol/L) is generally safe [83], which usually occurs when consuming a VLCKD that involves restricting CHO to 20–50 g/d (or <10% of daily kcal of 2000 kcal/day) [2,3,4]. Starvation ketosis during pregnancy is not a life-threatening condition and may cause a mild acidosis after a short-term overnight fasting [84]. Starvation ketosis occurs in pregnant women with pre-existing or gestational diabetes mellitus (GDM) when caloric intake is depleted [85,86]. Accelerated starvation contributes to diabetic ketoacidosis (DKA) in pregnancy and results in increased glucose utilization by both the fetus and the placenta, which leads to a rapid decrease in maternal fasting glucose levels. This decrease, along with insulin deficiency, stimulates the direct release of FFAs from AT, which are then circulated to the liver and converted to ketones [87]. DKA refers to the pathological state in which blood βOHB is higher than 3 mmol/L [88,89], and is generally unsafe during pregnancy [87,90,91,92]. DKA in pregnancy occurs as a consequence of metabolic and hormonal changes [87,93], and is characterized by uncontrolled hyperglycemia, ketonuria, ketonaemia and increased anion gap metabolic acidosis [85,87,90,91,92,93,94,95,96]. Women with gestational diabetes during the second and third trimesters are susceptible to ketosis due to increased insulin resistance and FFAs, which further enhance AT lipolysis as well as the hepatic ketogenesis process [93]. In this situation, even a short-term starvation precipitated DKA, which has adverse effects on both the mother and the fetus, including impaired intelligence and fetal demise, abdominal pain, weakness, nausea/vomiting, hyperventilation, hypotension, tachycardia, coma, polydipsia and weight loss [87,92,93,95,96,97]. Blood KB content has been shown to be higher in obese pregnant women with GDM compared with those without GDM, suggesting that obese GDM pregnant women may develop ketosis due to abnormal glucose metabolism [98]. An intervention through a low-carbohydrate-high-protein and fibre diet plus physical activity has resulted in increased neonatal FFAs in cord blood, and fasting glucose, FFAs and 3βOHB in obese women at 24–28 weeks of gestation, suggesting stimulation of white adipose tissue (WAT) lipolysis [99]. It can be suggested that the mild ketosis that occurs during starvation/fasting or feeding a VLCKD may enhance AT lipolysis and increase KB synthesis used as glucose substitutes to fuel in pregnant women with obesity/impaired glucose metabolism and newborn infants.

Under fasting conditions, plasma FFA, glucagon and βOHB levels are increased in lactating women, reflected in increased fat oxidation and lipolysis [100]. Lactation ketoacidosis (LK) is a relatively rare phenomenon of increased anion gap metabolic acidosis in postpartum lactating women during periods of acute illness [101], with few reported cases in situations of starvation, physical activity and KD found in the literature [101,102]. LK is considered a harmful form of starvation ketosis resulting from a raised glucagon-to-low-insulin ratio and subsequently increased KB production [86]. Adherence to KD for postpartum lactating women is associated with weight loss, vomiting, malaise, diarrhea, nausea, lower back cramps and abdominal pain [101,102].

## 4. Ketone Bodies and SCFAs as Epigenetic Modifiers in Obesity

Epigenetic changes constitute the key contributing factor of obesity during early development [47,103], in which heritable changes in gene expression result from histone modifications, DNA methylation and non-coding RNAs, without modifying the DNA sequence [104]. Genetic and/or environmental factors (e.g., nutritional changes, metabolic surgery, exercise) are thought to drive these epigenetic changes, in which several obesity-related traits, revealing cytosine-phosphate-guanine dinucleotides (CpG)-related sites (e.g., GNASAS1, MEG3, INSIGF2) are involved in altering DNA methylation in blood cells of the offspring [47]. Exposure to low a glycaemic index diet among obese pregnant women has been shown to induce DNA methylation changes at 771,484 CpG sites located in NFIC, TBCD and IL17D genes in the offspring cord blood [105]. Maternal obesity and high-fat intake during gestation may affect trans-generational epigenetic modifications. This is achieved through DNA methylation and chromatin alterations in adipogenic gene transcription, in which key epithelial to mesenchymal transcription (EMT)-related transcription factors (Slug, Zeb1, Zeb2, Snail, Twist) are involved, leading to increased obesity risk in the fetus [106]. Pre-and postnatal high-fat diets alter the gut microbiota in the offspring as well as DNA methylation and histone modification that result in changing adipogenesis-related gene expression such as adiponectin, leptin and peroxisome proliferator-activated receptor (PPAR-γ), leading to increase obesity and metabolic diseases later in life [107]. A few human studies investigating the epigenetic changes of early postnatal nutrition showed thatCpG3 methylation of leptin (LEP) and retinoid X receptor alpha (RXRA) obesity-related genes in infants are increased or decreased, depending on the duration of breastfeeding, and as a result, activate the PPAR-induced DNA demethylation in WAT, which drives changes in breast milk fatty acid (BM FA) composition [108]. A long-term folic acid supplementation of 400 μg/day (>6 months) and the dietary intake of betaine in pregnant and/or lactating women are shown to increase cord blood LEP and RXRA methylation in infants [109,110]. However, the impact of other dietary and supplemental methyl-group donors on these methylation changes have not yet been studied, given that methyl-donor intake through diet and supplementation may alter DNA methylation patterns in gene and disease susceptibility in humans [111,112].

Pregnancy and lactation are characterized by increased markers of OS and inflammation [52,53,54,55]. OS is induced by obesity in pregnancy, which may cause decreased fertility and increased miscarriage risk [54]. The OS markers, superoxide anion, nitric oxide, carbonyl proteins and malondialdehyde, have been observed in obese pregnant women, which leads to impact fetal redox balance [52]. The OS marker, 8-hydroxy-deoxyguanosine (8OHdG), along with lactose concentrations in breast milk, are found to be associated with a weight-for-length Z-score (WLZ) trajectory among infants of lactating overweight/obese women [53]. Increased breast milk inflammatory cytokines (IL-8, IL-6, and IL-1β) have been found to be associated with increased weight gain in infants [55]. The βOHB, a surrogate marker of liver ketogenesis, has been shown to regulate gene expression by inhibiting histone deacetylases (HDACs) and activating G-protein coupled receptors (GPCRs), and this may contribute to protection against OS and increased histone acetylation by inducing gene expression of Metallothionein 2 (Mt2) and forkhead box (Foxo3a) that encode oxidative stress resistance [113]. Under prolonged fasting, histone lysine β-hydroxybutyrylation (kbhb), a type of histone post-translational modification, which regulates gene expression, is increased in human embryonic kidney 293 (HEK293) cells as a result of βOHB level elevation [114,115]. Administration of βOHB on the human gut microbiota has been found to be associated with increased butyrate and SCFAs (sum of propionate, succinate, acetate, lactate and butyrate) production [116]. Butyrate promotes histone acetylation, inhibits HDACs activity in HEK293 cells, and suppresses lipopolysaccharide (LPS)-induced pro-inflammatory gene production in human adipose microvascular endothelial cells (HAMEC), including C-C motif chemokine ligand (CCL2), IL-6, IL-8, and IL-1β [115]. It has been shown that HEK293 cells are transiently transfect with the mutant melanocortin 4 receptor (MC4R) [117,118], a rhodopsin-like GPCR expressed in the hypothalamic pro-opiomelanocortin (POMC) neurons and the gene most commonly linked to obesity [119], which is located on chromosome 18q21.31at an early age [120]. MC4R mutant variant dysfunction may decrease ligand binding and expression of the receptor at the cell surface, with a reduction in MC4R agonist α-melanocyte-stimulating hormone (α-MSH)-induced cyclic adenosine monophosphate (cAMP) production, resulting in increased obesity and hyperphagia [117,118]. Leptin and insulin act on anorexigenic POMC neurons by signalling via its receptors to increase melanocortins and inhibit the orexigenic agouti related neuropeptide (AgRP)/neuropeptide Y (NPY) neurons, resulting in enhanced processing of POMC to α-MSH, decreased food intake and enhanced energy expenditure [121]. In diet-induced obesity, elevated activation of inflammatory pathways such as nuclear transcription factor-kappaB (NF-kB) and inhibitors of nuclear factor kappa-B kinase β (IKKβ) induce levels of suppressor of cytokine signaling-3 (Socs3) mRNA in POMC neurons and disrupt leptin/insulin signalling, leading to the development of insulin/leptin resistance in obesity [121,122]. SCFAs, and in particular acetate and propionate, influence intestinal epithelial cells through binding to FFA2/GPR43 and FFAR3/GPR41 expressed in AT in humans [123,124], leading to inhibition of signalling to the orexigenic hypothalamic neurons through systemic circulation by stimulating the secretion of key gut hormones, including glucagon-like peptide 1 (GLP-1) and peptide YY (PYY) [124,125,126], which indirectly regulate food intake and energy expenditure by increasing leptin and insulin secretion in adipocytes [124,125]. Taken together, βOHB and SCFAs act as potent epigenetic modifiers and exert anti-obesity effects providing a potential target in the treatment of obesity-induced inflammation and OS in children through interactions of leptin and insulin signalling in hypothalamic neurons, leading to regulated food intake and energy expenditure.

## 5. The Therapeutic Role of the Infant Gut Microbiota-Derived SCFAs in Obesity

This section presents the potential therapeutic target for gut microbiota-derived SCFAs in obesity. These include Gram-positive *Actinobacteria* (*Bifidobacterium* spp.), *Firmicutes (Lactobacillus* spp., *Streptococcus* *thermophilus*, *Blautia* spp.) and Gram-negative *Bacteroidetes* (*Bacteroides* spp.).

The *Firmicutes:Bacteroidetes* ratio (F/B) is identified as an important obesity-related biomarker. The obesity phenotype in human adults is associated with depleted levels of *Bacteroidetes* and an increased F/B ratio, which in turn leads to increased faecal butyrate, propionate, acetate and total SCFA concentrations [127,128]. In children and pregnant women, the association of obesity with the F/B ratio is unstable. Obese children exhibit lower numbers of *Bifidobacterium* and *Bacteroides* spp. [27,129], and higher numbers of *Staphylococcus aureus* (*S. aureus*) and *Fecalibacteriumprausntzi* (*F. prausntzi*) [129]. Lower numbers of *Bifidobacterium* and *Bacteroides*, and higher numbers of *S. aureus* and *Escherichia coli* (*E. coli*) are also detected in obese pregnant women [129]. Furthermore, there is evidence for the association between changes in milk microbiota and obesity development, although this association remains complex and little understood. Reduced levels of *Bacteroides* in milk microbiota are detected in obese mothers [130], who are exposed to a high fat and sugar diet during pregnancy and lactation [131].

### 5.1. Lactobacillus and Bifidobacterium spp.

*Lactobacillus* spp. mainly represented by *L. salivarius,* *L. fermentum*, *L. paracasei*, *L. reuteri,* *L. rhamnosus*, *L. casei* and *L. acidophilus* are the predominant LAB members of the breast-fed infant gut [132,133,134,135], which are demonstrated to have a high ability to produce bacteriocins and SCFAs [136,137]. *Bifidobacterium* spp. colonize the gut during the few weeks of life [138]. *Bifidobacterium* strains, such as those belonging to the species *B. longum* subsp. *B. bifidum* [132,133,139,140,141], and *B. breve* [133,142] represent the predominant members of breast milk, which produce acetate and lactate in the proportion of 3:2 during anaerobic CHO breakdown [16].

Probiotics are bacterial strains used to modulate the diversity and abundance of gut microbiota by increasing the production of SCFA-producing bacteria, including *Lactobacillus* and *Bifidobacterium* [143]. A small number of human RCTs showed that *Lactobacillus* and *Bifidobacterium* used as probiotics reduced obesity and metabolic disorders in pregnant and lactating women [124]. A review and meta-analysis which included seven RCTs showed that probiotic supplementation with *L. acidophilus*, *L. salivarius*, *L. casei* and *B. bifidum* strains among pregnant women with GDM resulted in significant reduction in infant birth weight [144]. Evidence from RCT studies supports the use of probiotic supplementation with *Lactobacillus* species in obese children, which results in reduced BMI, waist circumference, low density lipoprotein cholesterol (LDL-C), serum triglycerides (TG) and total oxidative stress levels [145,146].

*L. acidophilus* LA5 has been shown to reduce total cholesterol, LDL-C, a homeostasis model assessment of insulin resistance (HOMA-IR), fasting insulin concentration and postprandial glucose in obese women [147]. The combination of *L. acidophilus* and *L. casei* with phenolic compounds and a hypocaloric diet resulted in a significant reduction in weight and fat mass in overweight women [148]. *L. acidophilus* LA5 has been found to attenuate obesity in vitro as demonstrated by downregulating LPS-induced human cytokine (TNF-α, IL-8 and IL-10) production in hepatocyte cell-line (HepG2) cells [149], suggesting *L. acidophilus* may have the potential to enhance gut dysbiosis and reduce cytokine production in LPS, which may reduce obesity in infants. It has been found that administering the *L. casei* strain DN114001 to lactating mothers resulted in reduced pro-inflammatory cytokine TNF-α in the breast-fed infant [150]. The consumption of yogurt supplemented with strains specific *L. acidophilus* LA5 and *L. casei* DN001 could significantly reduce fat percentage, BMI and pro-inflammatory cytokines (IL-10, IL-17) produced by peripheral blood mononuclear cells (PBMCs) in obese individuals [151,152]. These strains have also been found to reduce leptin levels in obese individuals, which could lead to impaired T helper 17 (Th17) cell-mediated IL-10 and IL-17 production [152]. Given that *L. acidophilus* and *L. casei* have been identified as SCFA-producing bacteria, further RCTs are needed to explore their anti-obesity effects in early infancy via elucidating the mechanisms by which such bacteria could reduce the pro-inflammatory cytokines upregulated by leptin.

Supplementation with *L. salivarius* strains is considered safe for humans, which is used to treat several diseases [153]. Probiotic *L. salivarius* strain UCC 118 reduces BMI, but does not reduce fasting glucose or other metabolic parameters (e.g., C-peptide, lipids) in obese pregnant women [154]. The *L. salivarius* strain CECT5713 exerts potent probiotic attributes, which could rectify gut dysbiosis and increase fecal butyrate production in breastfed infants [155]. Butyrate exerts its anti-inflammatory functions by inhibiting HDAC activities in vitro and regulating GPR41 and GPR43-mediated hypothalamic neurons to control energy expenditure, resulting in stimulated “satiety” hormone PYY and GLP-1secretion from intestinal L cells via upregulation of Toll-like receptor(TLR)-dependent microbial sensing, and regulated leptin/insulin production from AT, and thus can be effective in reducing pro-inflammatory cytokines such as TNF-α and interleukins [24]. This suggests that butyrate-producing *L. salivarius* may be considered as a potential treatment for obesity in infants by reducing inflammatory cytokines and regulating leptin/insulin levels, but this requires further investigations and elucidation through future RCTs.

Probiotic supplementation with *L. rhamnosus* GG (LGG) 53103 one month before birth resulted in reduced weight in infants at the age of 2 and 4 years, but not later in life [156]. LGG 53103 supplementation has the ability to reduce weight in pregnant and lactating women when used in combination with *B. lactis* [157]. The *L. rhamnosus* strain CGMCC1.3724 in combination with caloric restriction resulted in reduced weight, fat mass and circulating leptin levels in obese women [158]. Early LGG colonization demonstrates anti-inflammatory activity by inhibiting *S. aureus* associated with increased numbers of cytokine producing cells at 2 weeks of age [159]. A novel secretory protein HM0539 derived from LGG has a potent protective effect against diseases related to intestinal barrier dysfunction in vitro, which enhance the development of the infant intestinal defence, as indicated by preventing against TNF-α or LPS-induced tight junction (TJ) protein expression, including intestinal mucin (MUC2) and zonula occludens-1 (ZO-1) [160]. High-fat diet-induced obesity enhances permeability in obese gut epithelium in vitro by activating pro-inflammatory cytokine signalling cascades induced by LPS stimulation through TJ protein expression upregulation [161,162], particularly claudin-2 [161]. Therefore, LGG may exhibit an inhibitory effect on LPS-induced pro-inflammatory cytokines via suppressing TJ protein expression in the gut epithelium. This suggests that LGG may inhibit TJ protein expression in the infant gut epithelia associated with obesity-induced inflammatory reprogramming, and thus, may exert anti-inflammatory effects in reducing obesity.

An experimental study has shown evidence that different strains of bifidobacteria isolated from infant feces including *B. breve* Bre10, *B. longum* BB536, *B. longum* Lon4 and *B. bifidum* Bif3 exert anti-inflammatory activity through suppressing LPS and TNF-α-induced IL-8 production and nuclear factor kappa B (NF-κB) activation when tested using the colon adenocarcinoma cell line (HT-29) cells [163]. These strains may inhibit LPS-induced pro-inflammatory cytokines in the infant gut [164], via increasing indole-3-lactic acid (ILA) production, a tryptophan metabolite, which functions as a ligand of the aryl hydrocarbon receptor (AhR) by enhancing nerve growth factor (NGF) induction in pheochromocytoma (PC12) cells through the rat sarcoma/extracellular signal-regulated kinase (Ras/ERK) signalling pathway [165]. An experimental study found that AHR signalling activation by a Western diet has a potential role in childhood obesity. Serum leptin, AHR and Cytochrome P450 family 1, subfamily B, polypeptide 1 (CYP1B1) gene expression levels are found to increase in obese children through inhibition of the AHR-aryl hydrocarbon receptor repressor (AHRR) [166]. This suggests that such strains may promote anti-obesity activity through increasing AhR ligand ILA production in the infant gut, and therefore, prevent transcription of pro-inflammatory cytokines. An RCT found that the administration of Inulin-type fructan (ITF) prebiotics to obese women led to a significant increase in the *B. longum* count, which in turn decreased serum LPS endotoxin production, thereby reducing pro-inflammatory cytokines production in AT [167]. Probiotic administration of *B. breve* BR03 and B632 strains to obese children resulted in reduced *E. coli* counts, systolic and diastolic blood pressure, waist circumference (WC), BMI, and enhanced insulin sensitivity at fasting and after an oral glucose tolerance test [168]. The administration of *B. breve* to preterm infants can upregulate transforming growth factor (TGF-β)-mediated deca-pentaplegic homolog3 (Smad3) phosphorylation [169]. In one experimental study, a suppression of the TGF-β/Smad3 pathway in WAT was shown to reduce obesity and regulate energy homeostasis/glucose tolerance in vitro. Smad3^−/−^ WAT in 3T3-L1 adipocytes exhibits elevated gene expression of brown AT (BAT)/mitochondrial markers such as Peroxisome proliferator-activated receptor-gamma coactivator-1alpha (PGC-1α). Exogenous TGF-β1 is able to inhibit the PGC-1α promoter in 3T3-L1 cells [170]. This result suggests that *B. breve* may promote regulatory TGF-β and reduce obesity in infants via inhibiting the TGF-β/Smad3 signalling pathway.

### 5.2. Streptococcus Thermophilus

*Streptococcus* spp. particularly *S. thermophilus* is able to produce L(+)-Lactic acid as the main CHO fermentation end product [171] and acetate from pyruvate via the acetyl-CoA and the Wood–Ljungdahl pathway of CO_2_ fixation, in which acetate is produced through the C_1_-body and the carbon monoxide methyl branches [172]. The *S. thermophilus* strain ATCC19258 metabilizesfucosylated human milk oligosaccharides (HMOs) (2′-FL or 3-FL) into lactate [173].

One RCT reported that a daily administration of *S. thermophilus* to overweight and obese children for 8 weeks did reduce BMI, LDL-C and TG [145]. A recent RCT has shown that supplementation of infant formula with prebiotic oligosaccharides, *B. breve* C50 and *S. thermophilus* O65 resulted in modulated gut microbiota and increased levels of secretory immunoglobulin A (SIgA) in the fecal contents of breastfed infants [174]. SIgA is capable of inhibiting pro-inflammatory cytokines associated with the pathogenic microorganisms in the intestinal epithelium. Following birth, specific IgA maternal antibodies have a direct effect on the infant’s gastrointestinal tract through its ability to associate with commensal microorganisms and enhance their immune function via the SIgA-specific receptor on the intestinal M cells [175]. TGF-β1 in colostrum has a significant effect on increasing SIgA production in infants [176]. One experimental study showed that non-colonized infants with Group B Streptococcus (GBS), a leading cause of infant mortality, were more likely to receive high colostral IgA antibody and low colostral TNF-α, IL-6 and IL-10 compared to colonized infants, suggesting that IgA antibody may contribute to reduced risk of GBS colonization and promote the maturation of the infant gut immune system [177]. Increased colonic fecal IgA levels in vitro was found to regulate obesity-related insulin resistance [178]. Intestinal B cells, T-cells and antibody-secreting cells (ASCs) have been shown to regulate obesity. Diet-induced obesity contributes to intestinal dysfunction, which is associated with reduced B cells and intestinal IgA^+^ ASCs and increased numbers of intestinal intra-epithelial CD8αβ^+^ T cells, leading to decreased SIgA levels. IgA^+^ ASCs may regulate AT inflammation by producing anti-inflammatory cytokines [179]. Therefore, it can be suggested that *S. thermophilus* may have a potential anti-obesogenic role in infants by producing SIgA, which has the potential to regulate AT inflammation.

### 5.3. Blautia spp.

*Blautia* spp. dominate the fecal microbiota of infants and breastfeeding mothers [180] and are known to be acetogenic bacteria among the hydrogenotrophic microbes present in the infant gut [181], which produce acetate from hydrogen and carbon dioxide through reductive acetogenesis using the Wood–Ljungdahl pathway, and the resulting acetate leads to the formation of butyrate [182].

*Blautia* spp. are included among SCFA-producing bacteria that showed a negative association with obesity in infants [28]. The relative abundance of *Blautia* is found to be significantly depleted in infants born to obese mothers [183]. A cross-sectional study has shown that pregnant women with GDM had a negative association between HOMA-IR, insulin and *Blautia* [184]. An experimental study showed that the depletion of *B. wexlerae* and *B. luti* spp. in the gut microbiota composition of obese children is associated with insulin resistance and increased production of pro-inflammatory cytokines and chemokines, including TNF-α, gamma interferon (IFN-γ) and MCP-1. The same study revealed that a high relative abundance of *Blautia* spp. in normal-weight children exerts anti-inflammatory effects on PBMCs by increasing the anti-inflammatory to pro-inflammatory cytokine ratios (IL4-TNF-α, IL4-FN-γ) [185]. PBMCs have been considered as representative of the alterations in inflammation-related adipokines, which may contribute to metabolic inflammation, leading to obesity and other related diseases [186]. Stimulation of the TLR signalling pathway, particularly TLR4 in obesity-induced inflammation by LPS in PBMCs may lead to increase pro-inflammatory cytokine production such as TNF-α and NF-κB [187]. PBMCs isolated from obese children were found to produce pro-inflammatory IL-1β levels and increases in microRNA33a and microRNA33b expression [188]. This suggests that *Blautia* spp. exerts anti-inflammatory effects on obesity by reducing inflammatory responses in obese AT.

### 5.4. Bacteroides spp.

*Bacteroides* spp. increase the production of acetate, propionate and butyrate as major end-products of fermentation of resistant starch in weaned infants′feces [189]. *Bacteroides* spp. produce propionate in the human gut through the succinate pathway, and acetate from pyruvate through the acetyl-CoA pathway [172].

A few studies have revealed that gut colonization with *Bacteroides* is significantly depleted in obese children [28,183,190,191] and pregnant women [184,192]. The relative abundance of *Bacteroides* spp. in infants may modulate immune response and gut inflammationby reducing the production of pro-inflammatory cytokines [26]. *B. fragilis* exerts anti-inflammatory activity in vitro as indicated by production of surface capsular polysaccharide A (PSA) in DCs, which stimulates the TLR2 signalling pathway as a mediator to induce CD39^+^ Foxp3^+^ regulatory T cells (T_regs_) with suppressed LPS-induced pro-inflammatory cytokine secretion [193]. In obese individuals, reduced numbers of circulating CD4^+^CD25^+^CD127^−^Foxp3 T_reg_ have been observed in PBMCs [194]. T_regs_ act as a subset of CD4^+^ T cells in human visceral AT that may result in activated anti-inflammatory mechanisms, where they may be involved in reducing the production of inflammatory cytokines associated with obesity [195]. A lack of T_regs_ in obese children leads to chronic low-grade systemic inflammation, glucose metabolic dysfunction and autoimmune diseases. In adaptive immunity, leptin exerts pro-inflammatory effects in AT through enhancing the proliferation of B and CD4^+^ T lymphocytes toward producing pro-inflammatory cytokines and reducing the production of anti-inflammatory Th2 cytokines [196].It is, therefore, suggested that *B. fragilis* may exert anti-inflammatory effects on AT in infants, due to their ability to modulate immune cell function and ameliorate AT inflammation. Further studies are needed to explore the mechanisms by which *B. fragilis* exert their anti-obesogenic effects in early childhood.

The gut commensal anaerobic *B. thetaiotaomicron* has been shown to regulate local immune responses and display potent anti-inflammatory activity to reduce pro-inflammatory cytokine expression in vitro by reducing nuclear export of NF-κB subunit relaxed (relA) aspartate-auxotrophic accumulation via a PPAR-γ-dependent pathway activation [197]. PPAR-γ activation has a beneficial effect on improving AT function and glycemic control/insulin sensitivity, reducing production of inflammatory mediators/macrophage-specific genes and preventing progression of metabolic disorders [51]. Thiazolidinediones are PPAR-γ agonists which regulate the expression of genes involved in reducing hepatic fat content and improving hepatic insulin sensitivity in vitro [51]. PPAR-γ concentrations have been found to be significantly lower in obese children than normal weight counterparts [198]. Inhibition of PPARγ expression in PBMCs is responsible for decreased angiopoietin-like protein 4 (ANGPTL4) levels in obese children, which play a role in reducing hepatic glucose production and promoting insulin sensitivity [199]. In one experimental study, reduced PPAR-γ expression in infant cell cultures was found to be associated with maternal obesity. Cells from infant of mothers with obesity showed mRNA (i.e., miR-1301, miR-221, miR-155) and PPAR-γ expression inhibition, and inflammatory cytokine (IL-6, IL-1β, TNF-α) expression upregulation [200]. This suggests that *B. thetaiotaomicron* may contribute to reducing obesity in infants by protecting AT against inflammatory cytokines by activating PPARγ expression.

**Figure 1:**
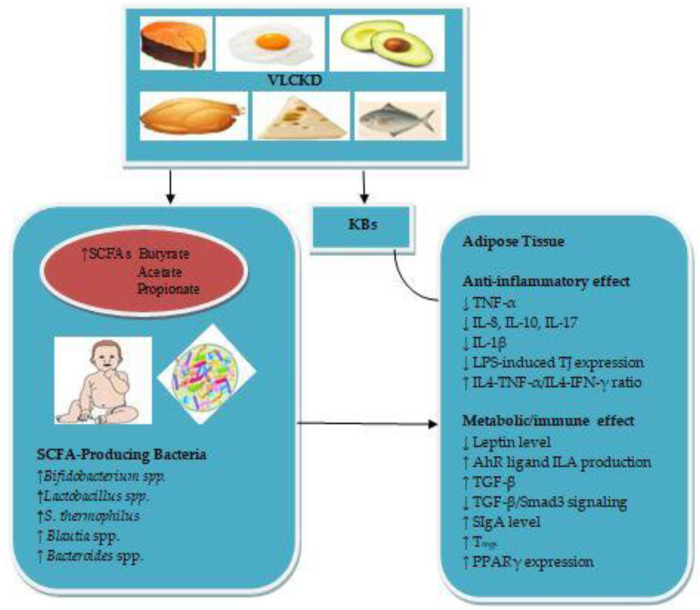
Prevention mechanism of Obesity.

The mechanisms by which the VLCKD during pregnancy and lactation may reduce obesity in infants are summarized in Figure 1.

## 6. Conclusions

The VLCKD has been proven effective as a restricted dietary pattern for treating obesity. However, its influence during pregnancy and lactation on SCFA-producing bacteria in infant gut microbiota and its mechanisms of action in the treatment of obesityare still unknown. Low CHO, high-fat and moderate-protein in a VLCKD regimen would be beneficial to maintain a continuous state of ketosis. Maintaining a nutritional ketosis is characterized by increased levels of ACA and βOHB in the blood, which are the main KBs that serve as energy sources during periods where CHO stores are reduced in the liver. The βOHB and SCFAs can influence epigenetic changes in infant obese gene expression and exert potential anti-obesity and anti-inflammatory effects by targeting obesity-associated inflammation via interactions of the hypothalamic appetite-regulating hormones leptin and insulin.

SCFAs appear to be the key microbial metabolites mediating VLCKD-infant gut microbiota relationships. Maternal VLCKD rich in PUFA, vegetable protein, dietary fibre, linoleic (ω-6) fatty acid and polyphenols stimulates the relative abundance of infant gut SCFA-producing bacteria, which may also utilize lactate as a substrate for SCFA production. SCFA-producing bacteria, including *Bifidobacterium* spp., *Lactobacillus* spp., *S. thermophilus*, *Blautia* spp. and *Bacteroides* spp. may be a potential treatment for obesity in infants. *Bifidobacterium* spp. exerts anti-inflammatory activity in ATby increasing AhR ligand ILA production, which may in turn lead to downregulation of pro-inflammatory cytokines. *Lactobacillus* spp. exerts an inhibitory effect on LPS-induced-pro-inflammatory cytokines in obese AT. *S. thermophilus* may regulate AT inflammation and enhance the maturation of the infant gut immune system via increasing SIgA production. *Blautia* spp. and *B. fragilis* exert anti-inflammatory effects on cytokine production in AT via regulating the TLR signalling pathway. *B. thetaiotaomicron* exerts anti-inflammatory effects in AT through activating PPARγ expression. Further studies would be needed to assess the safety of VLCKD during pregnancy and lactation to illuminate its potential influence on infant gut SCFA-producing bacteria. Further RCTs would also be needed to elucidate the mechanisms by which SCFA-producing bacteria exert their anti-inflammatory effects in AT in early childhood.

## Data Availability

Not applicable.
